# Phase II Study of Dose-Escalated and Convergent Stereotactic Body Radiotherapy for Liver and Pulmonary Oligometastases from Colorectal Cancer

**DOI:** 10.3390/cancers18081263

**Published:** 2026-04-16

**Authors:** Shuichi Nishimura, Atsuya Takeda, Yuichiro Tsurugai, Naoko Sanuki, Takahisa Eriguchi, Takafumi Nemoto

**Affiliations:** 1 Radiation Oncology Center, Ofuna Chuo Hospital, Kamakura-shi 247-0056, Kanagawa, Japantakatohoku@rad.med.keio.ac.jp (T.N.); 2 Department of Radiology, Keio University School of Medicine, Shinjuku-ku, Tokyo 160-8582, Japan; 3 Department of Radiation Oncology, Saitama Red Cross Hospital, Saitama-shi 330-0081, Saitama, Japan

**Keywords:** colorectal cancer, oligometastases, stereotactic body radiotherapy, SBRT, liver metastases, lung metastases, local control, radiation dose escalation, non-surgical treatment, toxicity

## Abstract

Purpose: Surgical resection of liver or pulmonary oligometastases (LP-OMD) in colorectal cancer (CRC) has been shown to improve survival. Stereotactic body radiotherapy (SBRT) is a promising alternative for patients with primary lung cancer. However, the efficacy of SBRT for LP-OMD in CRC remains inconclusive, and local control (LC) rates are often unsatisfactory. This prospective study aimed to evaluate the treatment outcomes of dose-escalated and convergent SBRT for patients with LP-OMD from CRC, with the goal of demonstrating its effectiveness as a treatment option for these patients. Methods and materials: This study included 23 CRC patients with LP-OMD who received SBRT between 2017 and 2022. The inclusion criteria were histologically confirmed colorectal adenocarcinoma, one to three oligometastases, and a tumor diameter of 5 cm or less. Patients who were inoperable or declined surgery were included. SBRT was delivered with total doses of 50–60 Gy administered over five fractions, covering the planning target volume surface within the 60% isodose line of the maximum dose. The primary endpoint was the 2-year LC rate, while secondary endpoints included overall survival (OS), progression-free survival (PFS), and toxicity. Results: The median follow-up duration was 41.0 months (range: 11.5–77.2). At the time of analysis, five patients had died from CRC, six were alive with disease, and twelve were alive without disease. Only one patient experienced local recurrence of a pulmonary oligometastasis. The 2-year LC, PFS, and OS rates were 95.0% (95% CI: 69.5–99.3), 61.3% (95% CI: 40.0–77.0), and 88.1% (95% CI: 67.6–96.0), respectively. Toxicity was acceptable, with no grade ≥ 3 adverse events. Conclusions: High-central-dose SBRT for LP-OMD from CRC achieved favorable local control with minimal toxicity. These findings should be interpreted cautiously and require validation in larger, multi-institutional studies.

## Simple Summary

Colorectal cancer can spread to the liver and lungs, and surgery is often the preferred treatment when possible. However, some patients cannot undergo surgery due to medical conditions or personal choice. In this study, we evaluated a specialized form of radiation therapy that delivers a higher dose to the center of tumors while minimizing damage to surrounding healthy tissue. We treated patients with a limited number of metastases and observed favorable tumor control with few side effects. These results suggest that this treatment approach may provide a safe and effective non-surgical option for selected patients. Further studies with larger patient groups are needed to confirm these findings and clarify its role in clinical practice.

## 1. Introduction

Approximately 15–30% of colorectal cancer (CRC) cases present with metastases at initial diagnosis, and an additional 20–50% develop metastases during follow-up [1]. The most common metastatic site is the liver, followed by the lungs [1,2].

In recent years, the advent of novel anticancer agents and molecular-targeted therapies has significantly improved survival in patients with stage IV CRC. As the survival of CRC patients treated with chemotherapy has increased, local therapy has become increasingly important for patients with oligometastases [3]. The National Comprehensive Cancer Network (NCCN) guidelines recommend resection of oligometastases in stage IV CRC patients with only liver or pulmonary oligometastases (LP-OMD) [4]. However, the feasibility of surgical intervention is often constrained by factors such as tumor location, residual liver function, patient age, and overall health. As a result, surgery is performed in only 10–30% of cases [5]. Furthermore, liver surgery carries complication rates of approximately 20%, with multiple reports indicating that such complications adversely affect prognosis [6,7,8].

Stereotactic body radiotherapy (SBRT) for primary early-stage lung cancer has demonstrated comparable efficacy to surgery in terms of LC rates and is a significantly less-invasive treatment [9]. It is now regarded as the standard of care for patients ineligible for surgery. Similarly, studies involving limited numbers of metastatic lesions from various malignancies have yielded encouraging outcomes with SBRT [10]. Thus, if SBRT achieves LC rates comparable to surgery, it may serve as a feasible treatment option for elderly patients and those with significant comorbidities.

We have administered SBRT to patients with pulmonary oligometastases; however, the LC rate for those originating from CRC was suboptimal in our cohort. This finding is consistent with a recent meta-analysis reporting inferior LC for CRC compared to non-CRC pulmonary metastases, possibly reflecting inherent radioresistance of CRC metastases [11,12]. Since 2011, we have treated patients with LP-OMD using SBRT with risk-adapted high-dose and convergent dose regimens, achieving a 100% two-year LC rate in a retrospective analysis [13]. Consequently, we designed this prospective study to validate the efficacy of this irradiation technique in treating LP-OMD from CRC.

## 2. Methods

### 2.1. Study Design

This was a prospective study involving patients recruited from Ofuna Chuo Hospital between March 2017 and September 2022. The study was conducted in accordance with the Declaration of Helsinki, and informed consent was obtained from all patients. The protocol was approved by our institutional review board (2016-017) and was registered with the University Hospital Medical Information Network (UMIN) (https://www.umin.ac.jp/ctr/, accessed on 13 April 2026; UMIN000026577). All patients were initially assessed for the feasibility of SBRT. Data analysis was performed in January 2025, ensuring that the interval between patient recruitment and data analysis exceeded two years for all participants.

### 2.2. Patients

Patients were eligible if they had oligometastatic disease, defined according to the ESTRO–EORTC consensus as a clinical state characterized by a limited number of metastatic lesions (typically ≤5), a controlled primary tumor, and the potential for curative-intent local ablative therapy [14]. For this study, oligometastatic disease was operationally defined as one to three metastases confined to the liver or lungs.

Inclusion criteria were:

- Histologically confirmed primary colorectal adenocarcinoma;
- LP-OMD confirmed by CT or MRI;
- One to three metastases with a maximum diameter ≤ 5 cm;
- Medically inoperable disease or refusal of surgery, as determined through multidisciplinary clinical assessment by a team including surgeons and oncologists;
- Eastern Cooperative Oncology Group (ECOG) performance status 0–2.

Additionally, PET-CT confirmed local control (LC) of the primary tumor and the absence of distant metastases to organs other than the liver and lungs. Patients were excluded if their treatment plans failed to meet the dose constraints specified in Table 1.

Concurrent chemotherapy was not allowed during SBRT. A minimum interval of two weeks was required between SBRT and chemotherapy, both before and after treatment.

In patients receiving systemic therapy prior to SBRT, the oligometastatic state was confirmed by the absence of disease progression for at least 3 months before treatment, in order to exclude rapidly progressive disease. In addition, in patients who received systemic therapy after SBRT, the oligometastatic state was retrospectively confirmed based on follow-up imaging, requiring the absence of early polymetastatic progression for at least 3 months after SBRT.

### 2.3. Treatment

A linear accelerator and a treatment planning system (Varian Medical Systems, Palo Alto, CA, USA) were used for treatment delivery and planning, respectively. Apart from the dose prescription, SBRT methods followed our conventional procedures described previously [15,16]. Briefly, we utilized a combination of vacuum-assisted cushions and abdominal corsets for patient immobilization. To visualize the internal target volume (ITV) directly, slow-scan CT imaging was conducted. No respiratory gating or active motion management was applied. A 6–8 mm expansion from the ITV was used to define the planning target volume (PTV).

Treatment planning employed volumetric modulated arc therapy (VMAT) with three arcs, using Eclipse version 4.2.0–4.3.3 (Varian Medical Systems, Palo Alto, CA, USA) and the Acuros XB algorithm with heterogeneity correction.

SBRT was delivered in risk-adapted total doses over five fractions based on metastasis location:

- 60 Gy in 5 fractions for peripheral pulmonary tumors without PTV–chest wall overlap and for liver metastases meeting dose constraints (central dose 100 Gy).
- 50 Gy in 5 fractions for peripheral pulmonary tumors with PTV–chest wall overlap, central pulmonary tumors, or liver metastases when 60 Gy could not be delivered while meeting constraints (central dose 83 Gy).

In all cases, the prescription dose covered the PTV surface with the 60% isodose line, creating a steep dose gradient and marked central dose escalation. This reflects a con-vergent dose strategy, in which a high central dose is intentionally concentrated within the tumor while respecting normal tissue constraints.

### 2.4. Follow-Up

Post-treatment surveillance included CT scans at 3- and 6-month intervals following SBRT, transitioning to a semi-annual schedule thereafter. MRI was used for liver metastases if appropriate.

For pulmonary metastases, physical examinations and blood tests were conducted monthly for the first 6 months, then every 3 months. For liver metastases, assessments were monthly for the first 3 months, then every 3 months.

### 2.5. Statistical Design and Sample Size Calculation

This study was designed as a single-stage prospective phase II trial. Based on prior reports [11], the threshold for the 2-year LC rate of SBRT for LP-OMD from CRC was set at 72%, with an expected rate of 90% for escalated-dose SBRT. Assuming a 3-year accrual period and a minimum follow-up of 2 years, with a power of 0.8 and a one-sided α of 0.05, the required sample size was calculated to be 18 patients. To account for potential dropouts, the target enrollment was set at 23 patients.

The primary endpoint was the 2-year LC rate, assessed on a per-lesion basis. Secondary endpoints included overall survival (OS), progression-free survival (PFS), toxicity, and patterns of failure. LC was defined as the absence of local progression at the treated site per RECIST v1.1 [17]. OS was calculated from registration to death from any cause; PFS included progression at the primary site, treated oligometastatic site, lymph nodes, distant metastases, or death. Toxicity was graded per CTCAE v4.0.

Kaplan–Meier methods were used to estimate survival outcomes, including OS, PFS, and LC. For LC and PFS, patients without events were censored at the date of the last fol-low-up imaging confirming no evidence of progression. For OS, patients were censored at the date of the last confirmed contact. Ninety-five percent confidence intervals (95% CIs) were calculated using standard methods. All statistical analyses were conducted using EZR (Saitama Medical Center, Jichi Medical University, Saitama, Japan), a user-friendly interface for R that provides a wide range of standard biostatistical tools [18].

## 3. Results

### 3.1. Eligible Patients and Tumors

Twenty-three patients were enrolled. Nineteen had pulmonary metastases (16 with a single nodule, 3 with two nodules), and four had liver metastases (3 with a single nodule, 1 with two nodules). No patients had simultaneous liver and lung metastases.

Sixteen patients had received chemotherapy prior to SBRT, with a median interval of 24 months from initial treatment to SBRT. Eleven patients received chemotherapy after SBRT. Detailed characteristics are shown in Table 2.

### 3.2. Local Control, Progression-Free Survival, and Overall Survival

**Figure 1:**
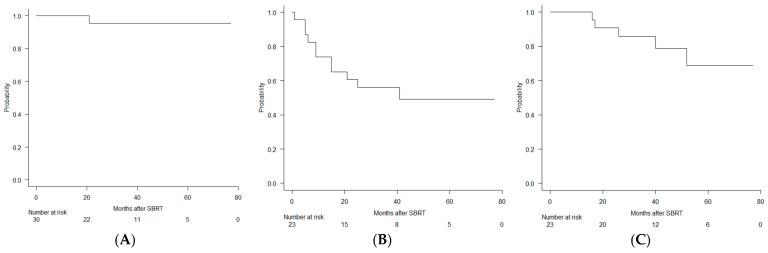
Outcomes after stereotactic body radiation therapy (SBRT) for limited liver and pulmonary oligometastatic disease from colorectal cancer. (**A**) Local control. (**B**) Progression-free survival. (**C**) Overall survival. Kaplan–Meier estimates are shown.

The median follow-up was 41.0 months (range: 11.5–77.2). The 2-year LC rate was 95.5% (95% CI: 71.9–99.3), meeting the expected primary endpoint (Figure 1A). The 2-year PFS was 60.9% (95% CI: 38.3–77.4), and the 2-year OS was 90.9% (95% CI: 68.3–97.6) (Figure 1B,C).

During follow-up, five patients died of CRC, while no deaths were attributable to other causes. At the time of analysis, six patients were alive with disease and twelve were alive without disease.

Only one local recurrence occurred, in a 15 mm pulmonary lesion treated with 50 Gy in 5 fractions, detected 23 months post-treatment. The patient subsequently received chemotherapy and was alive at the time of analysis.

### 3.3. Toxicities

No patients experienced grade ≥ 2 toxicities. All observed adverse events were limited to grade 1, including mild radiographic findings such as asymptomatic radiation pneumonitis or minimal liver function abnormalities, and no unexpected or late toxicities were identified during the follow-up period.

## 4. Discussion

This prospective study demonstrated the clinical utility of a risk-adapted, dose-escalated, and convergent SBRT regimen, achieving over 90% 2-year LC for LP-OMD from CRC. These findings support the effectiveness of our intensified SBRT approach in overcoming the traditionally suboptimal LC observed in CRC metastases. The results have significant clinical implications, showing that SBRT can serve as an effective local treatment option for patients who are not candidates for surgical resection due to tumor size, location, or general health status. To our knowledge, this is among the highest LC rates reported in clinical trials evaluating SBRT for LP-OMD from CRC [19,20].

SBRT may offer a viable treatment option even for patients with clinically unfavorable backgrounds. This non-invasive modality has shown promising efficacy and safety profiles, making it particularly suitable for patients ineligible for surgical resection or radiofrequency ablation (RFA). According to the NCCN guidelines, combined chemotherapy and surgical resection is recommended for LP-OMD from CRC [4]. When resection is feasible, 5-year OS rates range from 35% to 58% for liver metastases and 38% to 63% for pulmonary metastases [21,22]. However, in clinical practice, surgery is often not indicated due to tumor location, comorbidities, or patient preference. In our cohort, the multidisciplinary team recommended SBRT primarily for patients with limited cardiopulmonary reserve, prior extensive resections, or technically challenging tumor locations.

The randomized EORTC 40004 trial demonstrated that adding local therapies to systemic treatment improved long-term OS compared with systemic therapy alone, underscoring the importance of aggressive LC in oligometastatic CRC [23]. Furthermore, the recent COLLISION trial reported that in patients with small, resectable CRC liver metastases, minimally invasive approaches such as RFA achieved oncologic outcomes comparable to surgical resection while significantly reducing adverse events, supporting the role of non-surgical curative options [24]. In this context, SBRT, which is even less invasive than RFA, represents a promising alternative. For example, although Widder et al. reported retrospectively, their study showed that patients with pulmonary oligometastases ineligible for surgery but treated with SBRT achieved survival outcomes comparable to those undergoing resection [25]. This highlights the importance of individualized treatment optimization, balancing invasiveness, patient characteristics, and risk assessment.

A key feature of our trial is the demonstration that escalation of the central tumor dose can achieve high LC rates. The utility of SBRT for oligometastatic disease has been shown in clinical trials such as SABR-COMET [26]. However, several studies have indicated lower LC rates for CRC metastases compared to other histologies [11,27]. Increasing attention has recently been paid to the relationship between radiation dose and LC in SBRT for oligometastatic CRC, with evidence suggesting that higher doses may improve outcomes. For example, a biologically effective dose (BED_10_) ≥ 120 Gy has been associated with reduced recurrence [28], while BED_10_ ≥ 115 Gy has been linked to improved LC and OS [29]. Moreover, liver metastases are considered more radioresistant than pulmonary metastases, highlighting the need for site-specific dose optimization [30,31,32]. While our study included a higher proportion of lung metastases (n = 26) compared to liver (n = 4), the dose-escalated approach achieved excellent LC regardless of the metastatic site, although larger cohorts are needed to confirm the equivalence in liver-specific outcomes. One possible explanation for the favorable local control observed in this study is the biological effect of delivering a highly intensified dose to the tumor center through this treatment strategy. Colorectal cancer metastases are considered relatively radioresistant, and higher radiation doses may therefore be required to achieve durable tumor control. By prescribing the dose to a lower isodose line, our approach enables an increase in the maximum intratumoral dose while maintaining acceptable doses to the surrounding normal tissues. This inhomogeneous dose distribution may enhance tumor cell kill, particularly in hypoxic or treatment-resistant regions that are more likely to be located in the central portion of the tumor. Although the detailed radiobiological mechanisms remain to be fully elucidated, this strategy may represent a rational approach to overcoming the radioresistance of colorectal metastases.

While the value of dose escalation is well established, recent reports suggest that increasing the central tumor dose in SBRT can enhance LC in both primary lung cancer and metastatic liver tumors [33,34]. We have previously employed a treatment strategy with a high central dose, achieving favorable outcomes using 50–60 Gy in 5 fractions prescribed to the 60% isodose line, corresponding to a central dose of 83–100 Gy, for oligometastatic CRC [13]. The present prospective study adopted a similar prescription, and the results confirm the efficacy of this approach.

One of the principal advantages of SBRT over surgical resection is its minimally invasive nature, coupled with a lower incidence of adverse events and minimal impact on quality of life (QOL) [35,36]. The reported incidence of grade ≥ 3 toxicity after pulmonary SBRT ranges from 0% to 5.3% [37], while severe radiation-induced liver injury and gastrointestinal toxicities following SBRT for liver tumors are rare [19,38]. In our study, despite the delivery of high-dose SBRT, no severe treatment-related adverse events occurred. These findings are consistent with our previous Phase I trial [13] and earlier studies [15]. The consistent application of strict organ-specific dose constraints contributed to both high LC rates and treatment safety.

The combination of SBRT and chemotherapy has not yet demonstrated a definitive benefit for LC. Some reports suggest pre-SBRT chemotherapy may improve LC [30], whereas others indicate possible worsening [39], leaving the impact unclear. Similarly, the benefit of post-SBRT chemotherapy remains uncertain. In surgical oncology, conversion chemotherapy is recognized as effective for managing liver metastases, and while adjuvant chemotherapy is recommended in the NCCN guidelines, the JCOG0603 trial—evaluating postoperative chemotherapy after liver resection for CRC metastases—reported improved relapse-free survival but no significant OS benefit [40]. Thus, no international consensus exists, and further investigation into combined strategies involving SBRT and chemotherapy is warranted.

Future perspectives in optimizing local control may also involve further refinement of target definition and treatment monitoring. In this context, the integration of novel molecular imaging technologies may play an important role. For instance, recent research has demonstrated the feasibility of near-infrared fluorescence/Cerenkov luminescence dual-modality imaging, particularly for PD-L1 targeting [41]. This approach may provide superior tumor characterization compared to non-specific imaging. However, further clinical validation is required to determine its potential role in enhancing treatment planning and response assessment for SBRT in colorectal oligometastases.

This study has several limitations. First, the sample size was relatively small (n = 23), resulting in a wide confidence interval for the 2-year LC rate (95.5%; 95% CI: 71.9–99.3), and the findings should therefore be interpreted with caution despite exceeding the pre-defined threshold of 72%. Second, as a single-institution study, the potential for selection bias cannot be excluded. In addition, the proportion of liver metastases was lower than anticipated. Third, the single-arm design precluded direct comparisons with other treatment modalities, such as surgical resection or standard-dose SBRT. Finally, although patients were categorized into oligo-recurrence and oligo-progression, heterogeneity in systemic therapy before and after SBRT may have influenced survival outcomes. Furthermore, while we recognized the potential impact of these treatments, the limited sample size precluded robust subgroup analyses to evaluate the efficacy of specific chemotherapy regimens. This heterogeneity should be considered when interpreting the survival data. Larger, multi-institutional studies, ideally randomized controlled trials, are warranted to validate the efficacy and safety of high-central-dose SBRT and to establish its generalizability.

## 5. Conclusions

In this prospective study, high-central-dose SBRT achieved favorable local control and was safely delivered in patients with LP-OMD from CRC. This approach may represent a treatment option for patients who are not candidates for surgical resection. However, given the small sample size and single-institution design, these findings should be interpreted with caution. Further validation in larger, multi-institutional prospective studies is warranted to determine their generalizability.

## Figures and Tables

**Table 1:** OARs and PRVs Dose Constraints.

OAR	PRV Margin (mm)	Dose Constraint
Lung	-	V20 Gy < 20%
Liver	-	V30 Gy < 40%
Chest wall	-	V30 Gy < 70 mL
Portal vein, Bile duct	3	Dmax (0.5 mL) < 40 Gy
Spinal cord	3	Dmax (0.5 mL) <25 Gy
Esophagus	3	Dmax (1 mL) < 25 Gy
Gastrointestinal tract	3	Dmax (1 mL) < 25 Gy
Trachea, Bronchus	3	Dmax (0.5 mL) < 25 Gy
Great vessels	3	Dmax (0.5 mL) < 60 Gy
Pulmonary artery	3	Dmax (0.5 mL) < 40 Gy
Heart	3	Dmax (0.5 mL) < 50 Gy
Brachial plexus	3	Dmax (0.5 mL) < 30 Gy
Gallbladder	3	Dmax (0.5 mL) < 40 Gy
Skin	-	Dmax (0.5 mL) < 40 Gy

Abbreviations: OAR = Organ at risk; PRV = Planning risk volume.

**Table 2:** Patient and treatment characteristics.

Parameter	Value
Number of patients	23
Median age, years (range)	74 (46–86)
Sex, n (%)	
Male	14 (61%)
Female	9 (39%)
Total number of metastases	30
Lesion site, n (%)	
Lung	26 (87%)
Liver	4 (13%)
Disease status, n (%)	
Synchronous oligometastases	5 (17%)
Oligo-recurrence	16 (53%)
Oligo-progression	9 (30%)
Median follow-up from SBRT, months (range)	40 (11–77)
Median tumor size, mm (range)	12 (5–28)
PTV marginal dose, n (%)	
60 Gy/5fractions	12 (40%)
50 Gy/5fractions	18 (60%)
CEA level before SBRT, n (%)	
Normal (<5 ng/mL)	17 (74%)
Slightly elevated (5–10 ng/mL)	1 (4%)
Elevated (>10 ng/mL)	5 (22%)
Median time from diagnosis to SBRT, months (range)	24 (6–89)
Systemic therapy, n (%)	
Pre-SBRT chemotherapy	14 (61%)
Post-SBRT chemotherapy	9 (39%)

Abbreviations: PTV = planning target volume; SBRT: stereotactic body radiotherapy; CEA: carcinoembryonic antigen.

## Data Availability

The data presented in this study are not publicly available due to patient privacy concerns but are available from the corresponding author upon reasonable request and subject to institutional review board approval.
